# Where, when, and how to close dialysis access gaps: incorporation of geographic information system into data-driven planning: A commentary

**DOI:** 10.1186/s13584-025-00724-1

**Published:** 2025-11-26

**Authors:** Gina S. Lovasi, Richard Remigio

**Affiliations:** Dornsife School of Public Health, Urban Health Collaborative, Drexel University, Philadelphia, PA USA

**Keywords:** Geocode, Geographic, Surveillance, Dialysis, Disability

## Abstract

Barriers to timely and consistent dialysis care include characteristics at the patient, facility, and area levels. An article by Schroeder and colleagues uses national data spanning several thousand patients served by 67 dialysis centers across 6 districts of Israel, illustrating an approach that could be extended to other health care facilities within and beyond this national setting. The methods and findings demonstrate challenges and utility of incorporating geographic information systems (GIS) into data-driven planning to alleviate capacity constraints. Accurate estimation of distance and travel time from home to dialysis center rely on geocoding methods, road networks, time-varying transit and traffic congestion, and analytic treatment of statistical outliers. Analyses of where capacity gaps exist have potential to guide opening, relocation, or closure of health care facilities. Attention to identities and comorbidities within area-level resident populations can be used alongside data on whether dialysis centers are oversubscribed to inform scheduling. An example discussed was feasibility of offering a third Friday shift that extends into evening hours, which depends on religious observance patterns of patients and staff. Although not available in data used by Schroeder and colleagues, patient-level indication for supine treatment due to functional limitations could inform selection of equipment within dialysis centers, improving care quality. Our commentary highlights the potential of integrating GIS data to characterize where, when, and how dialysis care is available, which can likewise be used across other health care sites to ensure timely response to health emergencies and to accommodate the increasing chronic care needs of an aging population. Internationally, data needs and opportunities to improve population outcomes and equity will depend on the current state of care availability and on crises, whether collective (conflict, inclement weather) or personal (bereavement or other loss of social support, disruption of commuting modes).

## Main text

Life expectancy trends and leading causes of death globally shifted during the COVID-19 pandemic [1]. Diabetes and chronic kidney diseases (CKD) contributed to a recent decrease in life expectancy, reversing previous trends toward improvement [1]. Globally, countries vary in both the prevalence of kidney disease and capacities for treatment [2]. Some lower or lower-middle income countries, particularly within rural agricultural communities in Latin America [3] and South Asia [4], have observed a considerable increase in CKD prevalence but have gaps in capacity for adequate treatment [5]. In contrast, higher-income countries such as Israel and the United States offer national coverage for dialysis care (through National Health Insurance and Medicare, respectively) and have greater resources and capacities to meet related care needs across the life course [2].

Importantly, even for higher-income countries, there are within-country disparities in management of chronic health conditions including end-stage renal disease (ESRD), often concentrating the burden of disease among those with lower socioeconomic status and among ethnic minorities [6]. Barriers to timely and consistent dialysis care include characteristics at the patient, facility, and area levels [7], and geographic information systems (GIS) analyses can advance our insights into such barriers and guide actions to ameliorate them [8].

An article by Schroeder and colleagues uses national data spanning several thousand patients served by 67 dialysis centers across 6 districts of Israel, illustrating an approach that could be extended to other health care facilities within and beyond this national setting [9]. The authors use data that includes travel distance from the location of patient homes to dialysis centers, as well as utilization rates comparing actual use to estimated potential based on scheduling and station availability. The authors also leveraged national healthcare and registry data to enhance understanding of district-specific dialysis care across Israel using descriptive statistics of dialysis accessibility and utilization. Despite using data with many strengths, gaps are also identified, including the lack of patient-specific commute time to appointments, variable accuracy of patient addresses from national database linkages, the coarseness of district-level data which masks differences at the sub-district-level (e.g., neighborhood-level), and the need for consistent detailed information on patient functional status.

In this brief commentary, we discuss how the approach and findings demonstrate the challenges and potential utility of incorporating GIS approaches into data-driven planning to alleviate travel-time barriers and capacity constraints. We highlight ways to inform where, when, and how dialysis care is made available.

*Where: Estimating location and distance to inform geographic placement of care facilities*.

The author’s depiction of GIS as among “new and advanced computing technologies” [9] may seem an exaggeration given the volume of prior literature using these tools in reference to dialysis care [8], yet undoubtedly there are audiences for whom this is new. Those audiences require support in understanding the terminology and data quality challenges that arise [10], and particularly the potential for bias if errors are not randomly distributed with respect to geography.

Accurate estimation of location and travel distance relies on several components, with the potential to impact the validity of health study findings [11]. First, addresses that represent the home (origin) and dialysis center (destination) must be available and complete. This is a foundational prerequisite to estimate patient-specific travel distance approximated by a straight line. Additional data on road networks and infrastructure, built environments, and transit or congestion conditions can influence more relevant road-network travel distance and travel time. Even when such refinements are feasible, which is not always the case, systematic bias or random errors remain likely, and warrant attention [12]. Cautious interpretation or quantitative bias analyses [13] can contextualize likely effects of errors on travel-based estimates. Efforts to estimate travel time can scale up in the future using machine learning [14].

In many cases, using secondary data analyses for purposes beyond those originally motivating data collection can limit an investigator’s control of data quality and limit the transparency to understand how errors arise [15]. On the other hand, addresses collected for governmental, billing, or business purposes may have had more occasions to be scrutinized and corrected than addresses provided for the sole use in a research study. Nonetheless, there may be reasons for the acquired data to pass validation checks as valid addresses even when these do not represent the origin or the destination of a care-seeking trip. One way this can arise is due to provision of a PO Box or mailing location that does not correspond to the actual home or center [15]; however, in the Israeli context this problem may be minimal for both patient addresses (due to the requirement of proof of residency by the National Health Insurance for claims eligibility) and clinic addresses (due to required inspections to maintain licensure). When taking this study as a model for other settings, others may benefit from considering potential errors in the addresses themselves. For example, for some retail establishments in the US we noted in national databases that an abundance of establishments occur at the same address, possibly representing duplicate records for administrative purposes rather than the site of providing goods or services [16], and this is being particularly common for medical facilities [17].

Representing the addresses on digital maps and estimating the distance between them requires first geocoding the addresses. Geocoding methods vary in accuracy depending on the pre-processing and manual cleaning of the addresses, use of premium reference road networks, and approach to observations detected to be outliers [11].

Analyses of geocoded data, when accurate, have the potential to effectively guide the opening, relocation, or closure of health care facilities, but care is warranted for extreme values that may be unduly influential on analyses. Errors in the addresses obtained from the Israeli Ministry of the Interior database or inaccuracies in the geocoding process could have contributed to the 1.1% of patients excluded as outliers by Schroeder and colleagues due to a distance to treatment greater than 65.1 km [9].

The analyses presented by Schroeder and colleagues highlight differences between districts that suggest additional kidney care centers may be needed in the North District, where the travel distances are longest [9]. In contrast, the South District would have a less compelling case; however, we note that the percentage of data excluded as extreme outliers was greater in the South. If these all represent true travel distances greater than 65.1 km rather than location errors, the true contrast between North and South Districts would be smaller than the contrast as presented.

A final consideration on the planning for where to provide new medical facilities is the surrounding built environment [18, 19]. Consideration of complementary access to goods and services, as well as transportation infrastructure, could aid in the refinement, guiding decisions on where within a given district to place a new facility, relocate an existing facility, or act to prevent facility closure. Additionally, for facility planning and transportation infrastructure, it is essential to design and provide sufficient parking for staff and patients, establish ample patient drop-off and pickup zones, and identify major public transit hubs for convenient access and short travel distances among staff and patients [20, 21].

*When: Factoring in area resident and staff identities to optimize current and future shift schedules and adherence to appointments*.

Importantly, Schoeder and colleagues went beyond the presence or absence of a dialysis care center to also consider the number of potential patients who could be served [9], which depends on the scheduling and staffing of shifts throughout the week. The adequacy of the current schedule is evaluated based on utilization rate, and the authors further note that the need for services is expected to increase in future years due to population aging. Estimated comparisons of utilization with capacity implicitly assume that all centers and their stations are fully staffed and all dialysis medical equipment is fully operational at all times. Where this assumption does not hold, stations and staff may not be adequate to accommodate and service the estimated number of patients within dialysis centers without interruption.

Attention to spatial differences in comorbidities further highlights that the insufficiently met needs in the North District are expected to continue to increase due to a higher prevalence of diabetes mellitus among the area residents. Of those already engaged as patients, monitoring over time is needed due to disease progression, changing comorbidity profile, and even emerging treatment complications.

The attention to both current and future needs bring into focus a decision that area-based studies commonly face: which denominator to use when considering the concentration of health-relevant amenities. Options commonly include the land area, the number of area residents, or count of patients (or another identified persons actively spending time in the area or with access to a facility, which may overlap incompletely with the residential population). Geographic areas such as Israel’s districts have both a resident population and a patient population undergoing treatment for ESRD. The latter is most directly relevant to current utilization. However, the broader resident population can inform future planning, including assessment and development of the capacity to provide staffing and provide licensed care within clinics. A useful patient-level indicator that can inform current and future needs includes patient compliance with scheduled appointments at their respective clinic. This patient-specific indicator may serve as a proxy for socioeconomic and location-specific challenges associated with traveling to appointments. Multiple barriers to treatment adherence exist that include health care, disease-related, therapy-specific, and patient-specific factors [22], and to these the incorporation of GIS data expands our attention to consider difficulties and delays in transportation from home to the site of care.

In the study by Schoeder and colleagues [9], an interesting intersection arises between the scheduling of shifts and population identities (i.e., characteristics that link individuals within communities with cultural norms and practices that affect care-seeking and work restrictions, such as based on shared ancestry or religious affiliation). The authors note that a third shift can be scheduled during evening hours on Fridays in some areas with a sufficient Arab ethnic minority, whereas in predominately Jewish areas this would not align with religious observance practices. This seems to be an instance where the resident composition with respect to identity groups is especially relevant, as both the patient and staff identities would affect the feasibility of serving three full shifts on a Monday-Wednesday-Friday schedule.

Even as geographic patterns are considered, timing is important across multiple scales. At the smallest time unit are hourly considerations for staffing and patient care options, which are affected not only by practices noted above but also by daily patterns in the surrounding context such as traffic and transit barriers which can affect the reliability of reaching the clinic as scheduled. The use of more granular, localized GIS data may be ideal for better understanding sub-district-level (e.g., neighborhood-level) factors that influence travel distances and actual travel times. Additionally, although not readily available, the use of individual, self-reported, qualitative information on patient travel experiences, including hindrances and advantages, may be of great utility for appreciating accessibility differences across districts and, potentially, between clinics. The scheduling within days or weeks is highlighted above intersects with hours of the day, with the potential to inform the operational decisions of health care facilities in order to provide appropriately timed care. This adds to information about the clinical characteristics that can guide us to anticipate future population needs in the coming years and decades.

An additional temporal lens to consider in future work is the contrast between routine circumstances and the disruptions to care that can arise in a crisis, such as conflict [23] or inclement weather [24, 25]. Region-scaled disaster preparedness, particularly for climate-driven extreme events involving patients, clinics, and the kidney care community, is critical in minimizing disruptions and optimizing adherence to essential needs of care [26]. While more limited in scope, personal crises affect patients’ access to social support (e.g., via bereavement or health changes for household and family members) or to a working vehicle. To further build on this work, additional data sources could be linked and leveraged [27], which can then inform an understanding of how socio-political and environmental conditions, comorbidities, identities, behaviors, and preferences can shape the health-related needs and medical delivery of local communities and national populations.

*How: considering disability and quality improvement opportunities*

An identified gap in the data used by Schoeder and colleagues was the lack of patient-level indicators as to whether functional status limitations require supine position treatment. Less than half of dialysis care centers had stations to provide supine position treatment.

Mis-alignment of functional needs and nearby center equipment and capacities may be contributing to travel time. The data used were not suitable to assessing this as a factor in why some patients did not utilize the closest dialysis center to their home. If this was substantiated as a factor, this would then present an opportunity to reduce barriers to timely care and improve care quality that relies on better equipping existing centers, complementing the options described above to address where and when care is offered.

While Schoeder and colleagues note that inspection visits are required to maintain licensure in this context [9], there may be information from the inspection visits which could be integrated to inform future planning. Even among those locations that met the Israel Ministry of Health’s standards, there may be patterns that can be analyzed to provide insights into why utilization levels vary. Likewise, in other national contexts, there may be variation at the level of the health care facility [8] which contributes to patient behavior and chronic disease management outcomes, as well as to geographic disparities in these outcomes.

There are opportunities to extend the use of GIS for data-driven decisions to benefit other conditions and settings of care. Adherence barriers related to geography seen for dialysis care may be likewise relevant for recurrent visits to pharmacies [28], mental health care providers [29], oncologists [30], and rehabilitation services [31]. Beyond chronic disease, GIS is relevant particularly where patient outcomes depend on reaching care quickly. Examples highlighting travel time as a barrier across settings span the life course, from emergency obstetric care [32] to cholera treatment [33] to cardiac arrest [34].

## Conclusions

We highlight findings from a recent analysis integrating GIS to inform future planning of dialysis care centers [9]. We illustrate how area-, person-, and clinic-level data can be combined to inform where, when, and how dialysis care is provided. Throughout, there are opportunities to recognize the strengths of the data used and the potential for elaboration to further guide action, particularly by factoring in the comorbidities and identities of area residents, and the variation captured during licensure inspections that may be relevant to utilization across national settings.

Internationally, data needs and opportunities to improve population outcomes and equity will depend on the current state of care and on crises that are collective (e.g., conflict, inclement weather [24]) or more personal (e.g., disruption of social support, making transportation to care more difficult [7]). Schroeder and colleagues point us to an opportunity to make data-driven decisions on dialysis care in Israel [9]. Future research has the potential to extend this work by considering enhanced spatial granularity and incorporating additional characteristics of places and people, application to care across other chronic conditions, acute care needs, and extension to other geographic settings.

## Data Availability

No datasets were generated or analysed during the current study.

## Abbreviations

GIS: Geographic information system
